# Prevalence of regional and *whole-heart* viability in patients with myocardial akinesis consecutively enrolled from 4 US hospitals

**DOI:** 10.1186/1532-429X-18-S1-Q33

**Published:** 2016-01-27

**Authors:** Dina Labib, Han W Kim, Dipan J Shah, Afshin Farzaneh-Far, John Heitner, Igor Klem, Anna Lisa Crowley, Faisal Nabi, Michele Parker, Robert Judd, Raymond Kim

**Affiliations:** 1 grid.26009.3d0000000419367961Cardiovascular MR, Duke University, Durham, NC USA; 2 grid.185648.60000000121750319Cardiology, University of Illinois at Chicago, Chicago, IL USA; 3 grid.63368.380000000404450041Cardiology, Houston Methodist DeBakey Heart & Vascular Center, Houston, TX USA; 4 Cardiology, New York Methodist, Brooklyn, NY USA

## Background

The efficacy of current and novel heart failure therapies is likely related to viability that denotes the presence of cardiac reserve. Previous studies assessed viability mostly on a segmental rather than patient level, and included hypokinetic segments, which by definition−given residual contractile function−are viable. Additionally, studies have suggested that long-term survival is affected by the presence of viability in akinetic, not hypokinetic, segments. Therefore, we aimed to assess viability among patients with myocardial akinesis referred for cardiovascular MRI (CMR).

## Methods

Data analysis was performed on a cloud-based system that is currently receiving de-identified searchable data from reports with full DICOM datasets for 23,275 consecutive CMR exams performed at 4 U.S. hospitals from 2010-2014. At the time of abstract submission, 8,242 CMR datasets have been analyzed, and analysis of all 23,275 is expected by the end of 2015. All data fields were derived from reports that had been electronically signed by board-certified physicians with Level 3 CMR training. Each dataset included scores for wall motion and hyperenhancement (HE) on a standard 17 segments AHA model. We included only patients having one or more akinetic segments, defined as having no visible systolic increase in wall thickness. Patients with dyskinetic segments−showing systolic wall thinning−were also included. Segment viability was defined as HE less than 50% transmural, and "*whole-heart"* viability was defined as present if all 17 segments were deemed viable.

## Results

**Figure 1:**
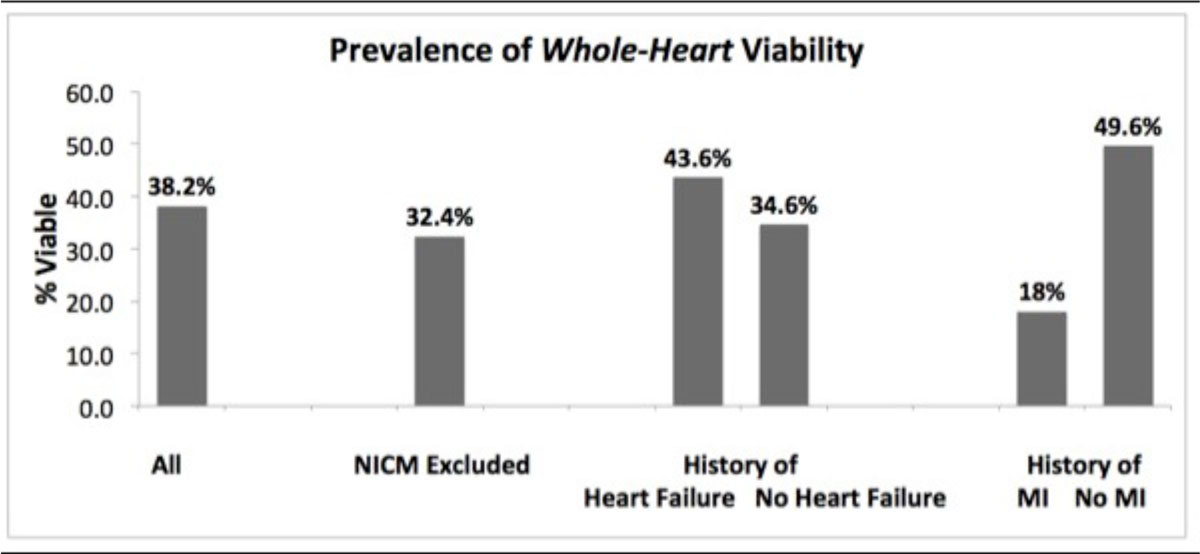
Figure 1

6,932 patients had cardiac scans with complete cine and delayed contrast enhancement imaging. Of these, 725 patients (10.5%) had myocardial akinesis of at least one segment. The table shows patient characteristics. Of 3091 akinetic segments, 37.6% were deemed viable. On a per patient basis, 38.2% (n = 277) had *whole-heart* viability. After excluding those with non-ischemic cardiomyopathy (NICM;15.9%), the prevalence of *whole-heart* viability remained high at 32.4% (n = 235). Other sub-cohorts are shown in the figure 1. The prevalence of *whole-heart* viability remained high in patients with severe left ventricular (LV) dysfunction (ejection fraction<30%) and in those with visible remodeling (severe LV enlargement), with prevalence of 45.3% and 50.0%, respectively.

## Conclusions

In this large multi-center study of patients with myocardial akinesis, over one-third of patients had *whole-heart* viability. The prevalence of *whole-heart* viability is high even in groups traditionally believed to have reduced viability, such as those with severe LV dysfunction, or remodeling, or history of heart failure. CMR identification of viability may identify patients who may benefit from existing and novel therapeutics for better design of heart failure trials.

**Table 1:** Characteristics in Patients with Myocardial Akinesis (n = 725)

Patient characteristic	Percentage
Age (mean ± SD)	56 ± 18 years
Male gender	61.2%
Diabetes mellitus	28.8%
Hypertension	60.2%
Hyperlipidemia	53.2%
Smoking	38.1%
Family history of CAD	36.3%
LV ejection fraction (mean ± SD)	35.1% ± 13.5%

